# Intention to use Medical Apps Among Older Adults in the Netherlands: Cross-Sectional Study

**DOI:** 10.2196/18080

**Published:** 2020-09-04

**Authors:** Marjan Askari, Nicky Sabine Klaver, Thimon Johannes van Gestel, Joris van de Klundert

**Affiliations:** 1 Erasmus School of Health Policy & Management Erasmus University Rotterdam Netherlands; 2 Prince Mohammad Bin Salman College of Business & Entrepreneurship King Abdullah Economic City Saudi Arabia

**Keywords:** Senior Technology Acceptance Model, intention to use, elderly, older adults, medical apps, mHealth, adoption

## Abstract

**Background:**

The increasing health service demand driven by the aging of the global population calls for the development of modes of health service delivery that are less human resource–intensive. Electronic health (eHealth) and medical apps are expected to play an important role in this development. Although evidence shows mobile medical apps might be effective in improving the care, self-management, self-efficacy, health-related behavior, and medication adherence of older adults, little is known about older adults’ intention to use these technologies when needed, or the factors influencing this intention.

**Objective:**

The objective of this study was to investigate the relationship of technology acceptance factors and intention to use mobile medical apps among community-dwelling older adults.

**Methods:**

Data was collected using questionnaires. The factors selected from the literature have been validated using Cronbach α and tested for significance using logistic regressions.

**Results:**

Almost half (49.7%) of the included older adults reported no intention to use medical apps. Adjusted logistic regression analysis per factor showed that the factors Attitude toward use (odds ratio [OR] 8.50), Perceived usefulness (OR 5.25), Perceived ease of use (OR 4.22), Service availability (OR 3.46), Sense of control (OR 3.40), Self-perceived effectiveness (OR 2.69), Facilities (OR 2.45), Personal innovativeness (OR 2.08), Social relationships (OR 1.79), Subjective norm (OR 1.48), and Feelings of anxiety (OR 0.62) significantly influenced the intention to use mobile medical apps among older adults, whereas the factor Finance (OR 0.98) did not. When considered together, a controlled multivariate logistic regression yielded high explained variances of 0.542 (Cox-Snell R^2^) and 0.728 (Nagelkerke R^2^).

**Conclusions:**

The high odds ratios and explained variance indicate that the factors associated with the intention to use medical apps are largely understood and the most important factors have been identified. To advance the evidence base, experimental controlled research should investigate the causality between the factors, intention to use, and actual use. For this purpose, our evidence suggests that policies designed to improve Attitude toward use appear most effective, followed by policies addressing Perceived usefulness, Perceived ease of use, Service availability, and Sense of control.

## Introduction

The number of adults over 65 years of age worldwide is expected to triple from 562 million in 2012 to 1.6 billion in 2050 and comprise 16.7% of the growing global population, up from the current 8.0% [1]. This aging of society is caused by increased longevity, decreased fertility, and the aging of the “baby boom” generation [1-3]. Older adults tend to make more use of health services compared to other age categories [4]. In the Netherlands, for instance, older adults form about 20% of the total population while accounting for approximately 80% of total health care expenditure [4]. The abovementioned three-fold global increase in the number of older adults may therefore indeed be expected to significantly increase global health services utilization and expenditure, exposing governments and societies to a wide range of social and economic challenges [5].

An important consequence of this aging-driven increase in health service needs is the additional demand for human resources it will create. For instance, the Dutch health care sector is expected to face a shortage of between 100,000 and 125,000 health care professionals by 2022 [6]. The largest shortages will involve nurses, geriatric specialists, and psychiatrists. Instead of resolving the increased health service needs by expanding the factor of production labor (ie, increasing human resources), policy makers are considering increasing human resource productivity, as facilitated by the factor of production technology [7]. Technology may, for instance, enable older adults to live in their own homes more independently and for a longer period of time [4].

Electronic health (eHealth), defined by the World Health Organization as the use of information and communication technologies for health, is widely considered to be a promising technological advancement to address the challenges presented above [8]. Its potential for health service delivery innovation and for service expansion without increasing human resource capacity is viewed as essential to addressing the increasing needs of the aging population with limited extra burden on the health care system [9].

Mobile medical apps provide an easily and widely accessible form of eHealth. Medical apps are defined as apps that run on electronic consumer devices such as smartphones and tablets [10-12]. These apps can, for example, be used to gather information about one’s health, disease, or condition; help monitor health; or support users in activities concerning their health [13-15]. Medical apps have been shown to improve the care, self-management, and self-efficacy of older adults, as well as promote better behavior and medication adherence [16-19].

While medical apps have been shown to be effective tools in supporting or substituting conventional health service delivery, evidence reveals that older adults tend to be more resistant to accepting new information technology apps [20,21] and to be apprehensive toward novel technologies [22]. In view of the aging-related challenges outlined above, and the contribution medical apps may have in resolving them, it is imperative to better understand medical app adoption by older adults. The widely accepted and validated Theory of Reasoned Action (TRA) and Theory of Planned Behavior (TPB), as well as more specific theoretic models introduced below, posit that medical app adoption is primarily determined by the intention to use such apps [23]. Our research aim is to advance the evidence base on factors influencing the intention to use medical apps among older adults.

The Technology Acceptance Model (TAM) derived from the TRA and TPB posits that Perceived ease of use, Perceived usefulness, and Social influence are the main factors of technology adoption, via the Intention to use factor [24,25]. Subsequent research has revealed a variety of additional factors influencing the intended and actual use of technology, such as Subjective norm, Voluntariness, Image (TAM2), Self-efficacy, Social norms, Trust, and Compatibility [16,25-29].

More recently, several studies have been conducted to advance understanding of the intention to use and actual use of medical technology [28,30-32] in general and by older adults in particular [23,33-35]. These studies, among which are studies specifically addressing intention to use medical apps, had quite small sample sizes [31,35] and/or took a qualitative approach [23,30,36]. Hence, while these studies have advanced toward a more specific Senior Technology Acceptance Model (STAM) [32], evidence on the factors determining intention to use medical apps by older adults is quite limited. Therefore, we set out to assess the validity and significance of factors determining intention to use medical apps in a quantitative study involving a large sample of older adults.

## Methods

### Study Design and Data Collection

A cross-sectional study was designed to study the relationship between the intention to use medical apps and proposed factors derived from literature. For this purpose, we developed a questionnaire and administered it both digitally as well as on paper to facilitate the inclusion of older adults with limited computer experience. Assistance and explanations were given to participants who needed help filling out the questionnaire, via telephone, email, or personal assistance when requested. The data was collected by 4 data assistants from November 2018 to June 2019 in cooperation with different types of organizations, such as living facilities and leisure activity clubs for older adults, general practitioners, and a hospital. To further strengthen data triangulation, we used online questionnaires, which were distributed across the Netherlands in cooperation with health service provider organizations and wellness organizations via different online channels and mailing lists. The reporting of the online questionnaire follows the CHERRIES checklist (Checklist for Reporting Results of Internet E-Surveys), which can be found in Multimedia Appendix 1 [37].

The inclusion criteria for participants were as follows: the participant is 65 years of age or older; the participant does not have cognitive impairments (as assessed by caregivers or those who distributed the questionnaires on paper); and the participant lives alone or with other people in a regular or senior living facility, the rationale being that older adults living in care facilities are already receiving care and therefore have little to no need for medical apps that promote time- and location-independent care.

At the start of the questionnaire, the following information was given in writing: the purpose of the project; information and instructions regarding the questionnaire; the expected duration of the survey, and the names of the main researchers. In addition, information about data management and privacy of the participants was provided to them. Before the participants filled out the questionnaire, an informed consent form was signed to give permission to use the data for research purposes. To alleviate potential concerns regarding privacy for the paper-based version, an envelope was provided to participants to ensure no one other than the data assistants would see the completed questionnaire. Data assistants entered the completed questionnaires into a SPSS database (IBM Corp) and pseudonymized the data to ensure anonymity.

### Senior Technology Acceptance Model

To analyze the association between acceptance factors and intention to use medical apps, an adapted and expanded version of the TAM for older adults is used. The TAM suggests that the Perceived usefulness and Perceived ease of use are key factors in explaining the intention to use, and subsequent use of a technological system [24,32]. We also included a number of factors from the STAM [32] and TAM2 [25], from which STAM is derived. In addition, we included specific acceptance factors for the use of medical apps among older adults from the literature, as indicated in Table 1, for a total of 12 factors. In addition, Table 1 shows the description of each factor and an example of a statement included in the questionnaire. For each factor, we included 1 to 4 statements to measure different aspects and strengths of the factor. These statements were answered using a 5-point Likert-scale (1=completely disagree, 2=disagree, 3=neutral, 4=agree, 5=completely agree). We computed a factor score by calculating the average score of all statements per factor.

The internal consistency of the items within the factors was investigated using Cronbach α [38,39]. The Cronbach α is expressed as a number between 0 and 1. The higher this number is, the lower the error variance is within the measuring instrument. The Cronbach α was acceptable if above 0.7 [40]. Items that, when removed, increased the Cronbach α of the acceptance factor by 0.1 or more were excluded from the acceptance factor and further analysis.

**Table 1:** Description of the included factors with an example statement and literature references.

Factor (number of statements)	Operational definition	Example of a statement	References
Perceived usefulness (3)	The extent to which a person believes that using the medical app will improve his or her quality of life	Using medical apps for remote health care would make my life easier.	[24,29,32,34]
Perceived ease of use (4)	The extent to which a person believes that using medical apps will be free of effort	It is easy to use medical apps for remote health care.	[24,25,29,32,34]
Attitude toward use (4)	An individual’s positive or negative feelings or appraisal about using medical apps	Using medical apps for remote care would be a good idea.	[24,29,32,34,41-43]
Subjective norm (3)	The person’s perception that most people who are important to them think they should or should not use medical apps	People who are important to me think that I should use medical apps.	[24,25,29,41-43]
Sense of control (2)	The perceptions of internal and external constraints on using medical apps	Using medical apps for remote health care is entirely within my control.	[29,42-44]
Feelings of anxiety (2)	An individual’s apprehension when he or she is faced with the possibility of using technology	I feel anxious to start using medical apps for remote health care.	[29,32]
Personal innovativeness (4)	Personal tendency to innovate, or introduce something new or different	In general, I do not hesitate to try out new information technology.	[20]
Social relationships (3)	An individual’s satisfaction with personal relationships and support from friends and family	I am satisfied with my personal relationships.	[32,45,46]
Self-perceived effectiveness (2)	Judgment of one’s ability to use medical apps to accomplish a particular job or task	I could perform a task on a medical app if I have just the instruction manual for assistance.	[29,32]
Service availability (3)	The obtainability and accessibility of medical apps	Medical apps for remote health care are always available whenever I need them.	[47]
Facilities (2)	Objective factors in the environment that can make technology usage easy. Included indicators are basic knowledge and available help	I have the knowledge needed to use medical apps.	[28,29,32]
Finance (1)	Having the financial resources to make technology usage easy	My financial situation stops me from using medical apps.	[28,29,32]

### Statistical Analyses

Descriptive statistics were used to analyze the composition of the research sample. For continuous variables, the mean and standard deviation were calculated; for categorical variables, percentages were used. The Assessment of Activities of Daily Living, Self-Care, and Independence (ADL) [48-50] and the Identification of Seniors at Risk – Primary Care (ISAR-PC) questionnaires [51,52] were used and scores were calculated as follows. ADL consists of 16 items and for every item, the participant answered whether they needed help doing the mentioned activities (such as showering, dressing, or walking). The ADL score was computed by counting the number of activities for which no help was needed [50]. The ISAR-PC was used to measure the increased risk of functional decline and consisted of three elements. The first two elements, household help required and repeated aptness to forget, are yes/no questions where answering with yes increased the ISAR-PC score by 2.5 and 2, respectively. The last element is an ordinal scale of 3 age groups: 65 to 74 years, 75 to 84 years, and 85 years or older. For individuals in the first group, the ISAR-PC score was increased by 0; it was increased by 1.5 for the second group and by 3 for the last group. An ISAR-PC score greater or equal to 2 shows an increased risk of functional decline [51].

An individual’s living situation was a categorical variable, consisting of 4 options: living independently alone, living independently with others, living in a senior living facility alone, or living in a senior living facility with others. Previous internet experience was a binary question. Perceived quality of life involved respondents rating their quality of life between 0 and 100. General health consisted of 5 categories: excellent, very good, good, fair, and poor. Health care utilization was the sum of the number of times a participant had visited the GP, the emergency department, and the hospital in the last 6 months.

### Univariate and Multivariate Logistic Regression

The calculated acceptance factor score served as input for the univariate and multivariate logistic regression analysis to examine the relationship between each of the acceptance factors as independent variables and intention to use medical apps as a dependent variable. Age, sex, and education served as control variables and were always included in the multivariate logistic regression model. Other candidate controls were marital status, living situation, ISAR-PC score, ADL score, previous internet experience, perceived quality of life, general health, and health care utilization. The controls that were measured on a continuous scale were tested on multicollinearity and one of two items that had an absolute correlation larger than 0.8 was removed, using expert opinion. The controls were iteratively added to the multivariate logistic regression model. Those that changed the odds ratio (OR) of any independent variable (the acceptance factor) by at least 10% when added to the multivariate logistic regression model were retained and incorporated in the final model [39,53]. Finally, we reported the measures of explained variance (the Cox-Snell *R^2^* and the Nagelkerke *R^2^*) for the multivariate logistic regression models.

### Validity and Reliability

To increase the internal validity, we included common, standardized, and validated instruments such as ADL and ISAR-PC [25,29,32,48,49,52]. Moreover, the questionnaire has been validated with the assistance of 4 older adults and several experts, including a geriatric nurse and 2 eHealth experts. The questionnaire is available on request. The database was checked for completeness and input errors, where a sample of paper questionnaires was compared to the database counterpart to check if they were identical.

To increase the external validity, we collected data for 40% of the respondents on paper, thus ensuring all eligible older adults were included. Moreover, data collection took place in several different geographical locations within The Netherlands. Lastly, Cronbach α was calculated for each factor to test the reliability. The study was approved by the medical ethical committee of Erasmus Medical Center (number MEC-2018-120).

## Results

### Population Characteristics

Our data set consisted of 364 older adults with an average age of 75 years (SD 7 years). Overall, 42.6% (n=155) of the 364 participants were male. Although 85.2% (n=310) of participants had experience using the internet, only 15.9% (n=58) had experience with medical apps. Despite the low proportion of participants that had ever used a medical app prior to filling out the questionnaire, more than 50.3% (n=183) stated an intent to use medical apps. Table 2 provides an overview of population characteristics.

A Cronbach α score was calculated for each of the acceptance factors to validate the internal consistency of the items within that factor [38]. The Cronbach α scores of the acceptance factors are shown in Table 3. All factors have an acceptable value of above 0.7 and none of the statements, when deleted, increased the Cronbach α by 0.1 or more [40].

**Table 2:** Baseline characteristics of the study cohort.

Characteristics		Participants (n=364)
Age (years), mean (SD)		74.9 (7.1)
Sex (male), n (%)		155 (42.6)
**Education, n (%)**		
	No education	9 (2.5)
	Lower education	57 (15.7)
	Intermediate education	160 (44.0)
	Higher education	125 (34.3)
**Marital status, n (%)**		
	Married	190 (52.2)
	Divorced	51 (14.0)
	Widowed	89 (24.5)
	Single	22 (6.0)
	Living with partner	8 (2.2)
**Living arrangement, n (%)**		
	Living independently, alone	129 (35.4)
	Living independently, with others	161 (44.2)
	Senior living facility, alone	34 (9.3)
	Senior living facility, with others	35 (9.6)
Identification of Seniors at Risk – Primary Care questionnaire score, mean (SD)		1.4 (1.7)
Assessment of Activities of Daily Living, Self-Care, and Independence score, mean (SD)		14.6 (2.3)
Quality of life, mean (SD)^a^		7.6 (7.0)
Prior experience with internet, n (%)		310 (85.2)
Prior experience with medical apps, n (%)		58 (15.9)
Intention to use, n (%)		183 (50.3)

^a^This measure is scored on a scale from 0 to 10.

**Table 3:** Cronbach α of the technology acceptance factors.

Factors^a^	Cronbach α
Perceived usefulness (n=3)	0.922
Perceived ease of use (n=4)	0.950
Attitude toward use (n=4)	0.955
Subjective norm (n=3)	0.974
Sense of control (n=2)	0.890
Intention to use (n=3)	0.969
Feelings of anxiety (n=2)	0.913
Personal innovativeness (n=4)	0.950
Social relationships (n=3)	0.716
Self-perceived effectiveness (n=2)	0.742
Service availability (n=3)	0.923
Facilities (n=2)	0.746
Finance (n=1)	N/A

^a^The n value refers to the number of statements within a construct.

### Univariate and Multivariate Analysis

To analyze the relationship between each acceptance factor and intention to use medical apps, univariate and multivariate analyses were performed. The results of the univariate logistic regression analysis showed that all factors were significantly associated with Intention to use medical apps, except for Finance. As expected, these results showed all factors to be positively associated with Intention to use, except for the factor Feelings of anxiety.

The multivariate logistic regression analyses largely confirmed the results of the univariate analysis. None of the controls displayed multicollinearity and therefore none of the variables had to be excluded in the multivariate logistic regression. Controlling for age, sex, and education modestly reduces or increases the ORs. The other candidate controls mainly impact the original TAM key factor Attitude toward use. Table 4 presents these results.

The two right-most columns of Table 4 present two measures of explained variance (the Cox-Snell *R^2^* and the Nagelkerke *R^2^*) for the models, which control for age, sex, education, and controls increasing the OR by more than 10%. When including all factors and none of the controls, the explained variances are 0.486 (Cox-Snell *R^2^*) and 0.651 (Nagelkerke *R^2^*). Adding the standard controls age, sex, and education did not have much of an impact on the explained variance. When adding all controls to the model, which includes all factors, the explained variances increase to 0.542 (Cox-Snell *R^2^*) and 0.728 (Nagelkerke *R^2^*), respectively.

**Table 4:** Association between acceptance factors and intention to use medical apps.

Factors	Univariate OR^a^ (95% CI)	*P* value	Multivariate OR (95% CI)^b^	*P* value	Multivariate OR (95% CI)^c^	*P* value	Included controls	Cox-Snell *R*^2^	Nagelkerke *R*^2^
Perceived usefulness	5.94 (3.88-9.08)	<.001	5.25 (3.41-8.07)	<.001	—	—	—	.340	.456
Perceived ease of use	4.43 (3.01-6.52)	<.001	4.22 (2.78-6.40)	<.001	4.71 (3.02-7.36)	<.001	ISAR-PC^d^	.298	.400
Attitude toward use	9.19 (5.52-15.30)	<.001	8.50 (5.03-14.38)	<.001	11.24 (6.08-20.79)	<.001	ISAR-PC, ADL^e^, marital state, health care use	.440	.591
Subjective norm	1.47 (1.17-1.84)	.001	1.48 (1.15-1.90)	.002	—	—	—	.129	.173
Sense of control	3.59 (2.64-4.87)	<.001	3.40 (2.45-4.72)	<.001	—	—	—	.293	.394
Feelings of anxiety	0.56 (0.44-0.70)	<.001	0.62 (0.47-0.81)	.001	—	—	—	.135	.181
Personal innovativeness	2.38 (1.85-3.06)	<.001	2.08 (1.58-2.73)	<.001	—	—	—	.182	.243
Social relationships	1.76 (1.21-2.56)	.003	1.79 (1.18-2.71)	.006	—	—	—	.131	.175
Self-perceived effectiveness	2.84 (2.10-3.82)	<.001	2.69 (1.93-3.76)	<.001	3.05 (2.14-4.36)	<.001	Living situation	.240	.321
Service availability	3.71 (2.61-5.26)	<.001	3.46 (2.37-5.06)	<.001	—	—	—	.245	.329
Facilities	2.70 (2.03-3.59)	<.001	2.45 (1.78-3.35)	<.001	—	—	—	.178	.239
Finance	0.93 (0.74-1.16)	.51	0.98 (0.76-1.28)	.90	—	—	—	.097	.129

^a^OR: odds ratio.

^b^Adjusted for age, sex, and education.

^c^Adjusted for age, sex, and education and all controls that increase the OR by at least 10%.

^d^ISAR-PC: Identification of Seniors at Risk – Primary Care.

^e^ADL: Assessment of Activities of Daily Living, Self-Care, and Independence.

## Discussion

### Principal Results

In this study, we aimed to provide the first robust quantitative evidence on the intention to use medical apps among community-dwelling older adults and the factors identified in the literature that could assist in determining intention to use. We found that almost half of the respondents (49.7%, n=181) had no intention to use medical apps. This first descriptive finding is relevant due to the important contribution medical apps are anticipated to make in keeping health services affordable as the number of older people worldwide triples, resulting in an increase in health service demand. Hence, it is important to understand the factors determining the intention to use medical apps. Here, we first synthesize the evidence as identified in our study results.

All but one of the proposed factors were very significantly related to Intention to use, with *P* values <.01. More specifically, the multivariate logistic regression analyses showed that the following acceptance factors are significantly related to the intention to use medical apps in this population: Perceived usefulness, Perceived ease of use, Attitude toward use, Subjective norm, Sense of control, Feelings of anxiety, Personal innovativeness, Social relationships, Self-perceived effectiveness, Service availability, and Facilities. All these factors were positively associated with Intention to use, except for the factor Feelings of anxiety, which was negatively associated with Intention to use. Finance was the only identified factor not significantly related to Intention to use. As our results showed, having feelings of anxiety about using new technology may negatively affect the intention to use medical apps. This might be caused by factors such as a lack of self-efficacy, a desire for a greater sense of control, privacy issues, or a lack of trust. Future studies are needed to study the underlying causal factors.

The factor Attitude toward use stood out with an OR of 8.50 in the multivariate model (11.24 when controlled), indicating that a positive Attitude toward use roughly increases the Intention to use ten-fold. The other two original TAM factors, Perceived usefulness and Perceived ease of use, had the second- and third-highest ORs, albeit much lower than that for Attitude toward use. Sense of control is another classic factor [29,42-44] that has an OR above 3; the same is true for the lesser known factor Service availability [47]. The lack of significance of the factor Finance, originally included in STAM [32] and confirmed in subsequent studies [28,29], might be explained by the relatively low cost of medical apps compared to other technologies and the relatively generous mandatory health insurance coverage in The Netherlands.

The variables ISAR-PC, ADL, marital status, and recent health care use significantly impacted the Attitude toward use. Otherwise, the added controls had little (<10%) effect on ORs, with only two exceptions (Table 4). This indicates that, perhaps with the exception of Attitude toward use, measures to improve factor scores do not need to distinguish among subpopulations but can target the entire population of older adults. Standing out for its high OR, the factor Attitude toward use appears to be a prime candidate for interventions to increase intention to use among older adults by making attitudes more favorable.

### Comparison with Prior Work

All the technology acceptance factors considered in this study are taken from the literature and have been positively associated with intention to use various forms of (medical) technology in other contexts, sometimes specifically for older adults [23,28,30-35]. Our research confirms the validity of these factors in explaining older Dutch adults’ intention to use medical apps.

Our findings are akin to the findings that Cajita et al [23] obtained for older adults with heart failure. They found Perceived usefulness, Perceived ease of use, Subjective norm, Social relationships, and Social influence to be significantly related to intention to use mobile technology [23]. These similarities arise despite the differences in patient populations, which include individuals from different countries, who have different morbidity and are of different sizes. Moreover, our findings strongly confirm the three original TAM factors as the main factors driving intention to use medical apps among older adults [24]. This robust finding is notable as these factors date back more than 30 years and technology has advanced considerably. Medical apps did not exist when TAM was developed. It is possible that the aging population has simply carried the factors associated with their generation into the future since 1989. In addition, our analyses confirm all but one of the factors of the recent STAM [32]. Altogether, these similarities suggest that our main findings have validity outside the Netherlands and beyond a near-term horizon.

### Limitations

A first limitation of our study may be the length of the questionnaire used. Although steps were taken to minimize the impact of the length of the questionnaire, such as printing out the questionnaire so participants could take breaks or sitting with the participants while they filled out the questionnaire, some participants still showed signs of response fatigue [54]. To minimize the impact of response fatigue, participants could take breaks and save their answers online to continue later on. Second, we noticed that some of the participants, especially those aged >75 years, struggled to understand the use and utility of medical apps. To address this situation, the questionnaires and interviewers provided additional explanations about medical apps. Due to the cross-sectional design of this study, no claims of causality can be made [55] and the results might suffer from self-report bias [56]. Lastly, while the data was collected from a variety of contexts in The Netherlands, we cannot claim validity in other countries, where for instance the factor Finance may be of larger significance.

### Recommendations and Future Research

The main contribution of this study is to provide the first large-scale quantitative evidence of the relationships between the proposed acceptance factors and the intention to use medical apps among older adults in the Netherlands. As noted, due to the research design, we cannot confirm causality among the identified relationships. Hence, a first recommendation is to advance research on the most significant factors using controlled experiments rather than large-scale cohort studies to confirm or refute any potential causality of the relationships found. Such studies may target older adults who do not yet intend to use medical apps; this is the most urgent group to include in such initiatives in view of the challenges related to the aging of societies. Moreover, even though behavioral intention has been shown to predict actual technology adoption [57], such experiments might study actual technology use, rather than intention to use. In addition, we recommend qualitative research to advance understanding regarding the nature of the relationships between the most significant factors and the intention to use. Meanwhile, policy designed at improving Attitude toward use appear most effective, accompanied by policies addressing Perceived usefulness and Perceived ease of use, as well as Service availability and Sense of control.

## Appendix

Multimedia Appendix 1 CHERRIES checklist.

## Abbreviations

ADL: Activities of Daily Living
CHERRIES: Checklist for Reporting Results of Internet E-Surveys
eHealth: electronic health
ISAR-PC: Identification of Seniors at Risk – Primary Care
mHealth: mobile health
OR: odds ratio
STAM: Senior Technology Acceptance Model
TAM: Technology Acceptance Model
